# The Mechanism of Flex‐Activation in Mechanophores Revealed By Quantum Chemistry

**DOI:** 10.1002/cphc.202000739

**Published:** 2020-10-07

**Authors:** Lennart J. Mier, Gheorghe Adam, Sourabh Kumar, Tim Stauch

**Affiliations:** ^1^ University of Bremen Institute for Physical and Theoretical Chemistry Leobener Straße NW2 D-28359 Bremen Germany; ^2^ University of Bremen Bremen Center for Computational Materials Science Am Fallturm 1 D-28359 Bremen Germany; ^3^ University of Bremen MAPEX Center for Materials and Processes Bibliothekstraße 1 D-28359 Bremen Germany; ^4^ Current address: University of Bremen, UFT Leobener Str. 6 D-28359 Bremen Germany

**Keywords:** Computational Chemistry, Density Functional Theory, Flex-Activated Mechanophores, Mechanochemistry, Quantum Chemistry

## Abstract

Flex‐activated mechanophores can be used for small‐molecule release in polymers under tension by rupture of covalent bonds that are orthogonal to the polymer main chain. Using static and dynamic quantum chemical methods, we here juxtapose three different mechanical deformation modes in flex‐activated mechanophores (end‐to‐end stretching, direct pulling of the scissile bonds, bond angle bendings) with the aim of proposing ways to optimize the efficiency of flex‐activation in experiments. It is found that end‐to‐end stretching, which is a traditional approach to activate mechanophores in polymers, does not trigger flex‐activation, whereas direct pulling of the scissile bonds or displacement of adjacent bond angles are efficient methods to achieve this goal. Based on the structural, energetic and electronic effects responsible for these observations, we propose ways of weakening the scissile bonds experimentally to increase the efficiency of flex‐activation.

## Graphical abstract

**Different deformations**: Using state‐of‐the‐art Density Functional Theory and computational strain analysis, it is shown that retro‐[4+2] cycloaddition in flex‐activated mechanophores in polymers cannot be initiated by end‐to‐end stretching of the polymer main chain. By contrast, the bending of bond angles in the polymer chain adjacent to the mechanophore is appropriate for this task, leading to the release of small molecules from mechanically deformed polymers.

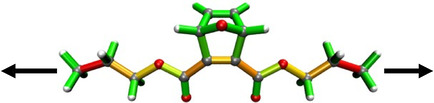

During recent years, the field of polymer mechanochemistry[^1^, ^2^, ^3^, ^4^, ^5^, ^6^] has matured to a point where a plethora of useful applications are available. Examples include the optical sensing of stress and strain using mechanochromic materials,[^7^, ^8^, ^9^, ^10^, ^11^, ^12^, ^13^, ^14^, ^15^, ^16^, ^17^] the self‐healing of polymers,[^18^, ^19^, ^20^] and the force‐induced activation of latent catalysts.[^21^, ^22^, ^23^] Many of today's functional polymers incorporate mechanophores, which are small subunits of the polymer that respond to mechanical deformation by significant structural changes and fulfil diverse tasks upon activation. One particularly intriguing class of these subunits, so‐called flex‐activated mechanophores,[^24^, ^25^] display the unique feature that bonds that are orthogonal to the polymer main chain are ruptured upon mechanical deformation. With this approach, small molecules can be released well before material failure occurs,^[26]^ which is helpful in the development of stress‐sensing and self‐healing materials.

However, the mechanism of flex‐activation has hitherto not been understood in detail. Considering that the efficiency of flex‐activation commonly lies below 10 %,[^24^, ^25^] detailed insights into the activation mechanism are direly needed, since this knowledge would allow the optimization of this process. In this study, the mechanism of flex‐activation in mechanophores is elucidated using quantum mechanochemical methods,[^27^, ^28^, ^29^] which have been used successfully in the past to, e. g., study the debated cycloreversion of triazoles,[^30^, ^31^, ^32^] develop novel molecular subunits for stress‐sensing in polymers,[^33^, ^34^, ^35^, ^36^, ^37^] and investigate the impact of topological effects on mechanical properties and the reactivity of molecules.[^38^, ^39^, ^40^]

The model system considered in this study (Scheme 1) is similar to the one examined in ref. 24, however, it includes longer alkyl chains to simulate the influence of the polymer surrounding more realistically. Successful activation of the mechanophore leads to the release of furan *via* a retro‐[4+2] cycloaddition. To the best of the authors’ knowledge, successful flex‐activation in experiments has only been described for systems that are very closely related to the model system considered here. Three different modes of deformation are applied, in particular end‐to‐end pulling (mode 1), direct pulling of the “critical” bonds that rupture upon successful activation of the mechanophore (mode 2) and displacements of the bond angles adjacent to the functional subunit (mode 3). Deformation modes 1 and 2 are attained *via* the External Force is Explicitly Included (EFEI) approach,[^41^, ^42^, ^43^] which is a quantum chemical geometry optimization under constant external force, whereas the COnstrained Geometries simulate External Forces (COGEF) approach,[^44^, ^45^] a geometry optimization with geometric constraints, is used for deformation mode 3. Each of the three deformation modes was applied progressively until rupture of a carbon‐carbon bond occurred. Density Functional Theory (DFT)[^46^, ^47^] at the PBE^[48]^/cc‐pVDZ^[49]^ level of theory is applied throughout. The full set of computational details is given in the Supporting Information.

**Scheme 1:**
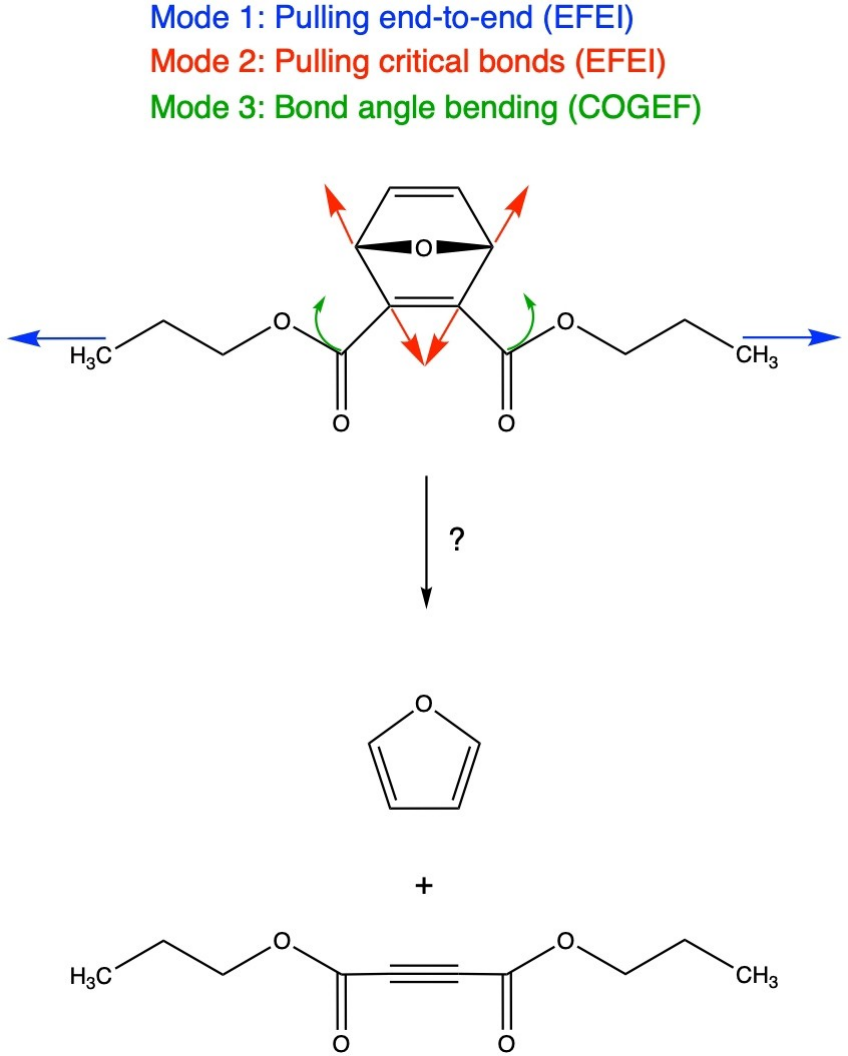
Deformation schemes of the model system considered in this work that might lead to the release of furan *via* a retro‐[4+2] cycloaddition.

Mechanical stretching of a polymer is intuitively viewed as an end‐to‐end stretching of the polymer chains. Applying this deformation to the model system (mode 1) leads to a rupture force of 5.9 nN, but, surprisingly, rupture occurs in the terminal carbon‐carbon bonds of the polymer main chain and not in the “critical bonds” that must be broken for a successful retro‐[4+2] cycloaddition. The reason for this effect is revealed by the Judgement of Energy DIstribution (JEDI) analysis,[^50^, ^51^, ^52^] which is a quantum chemical tool to analyze the distribution of strain energy among the various bonds, bendings and torsions of a mechanically deformed molecule (Figure 1). The critical bonds are hardly elongated and therefore do not store significant amounts of strain energy. Instead, the terminal carbon‐carbon bonds that connect the methyl groups to the rest of the polymer linker store increasing amounts of strain energy with increasing pulling forces. While several other bonds and bendings in the polymer chain are also being strained during elongation of the molecule (cf. the color‐coded structures in Figure 1), bond scission ultimately occurs in the terminal carbon‐carbon bonds of the polymer chain. Hence, a simple stretching of the polymer main chain, e. g. in mechanical pulling experiments, is insufficient to trigger small‐molecule release in this flex‐activated mechanophore.

**Figure 1:**
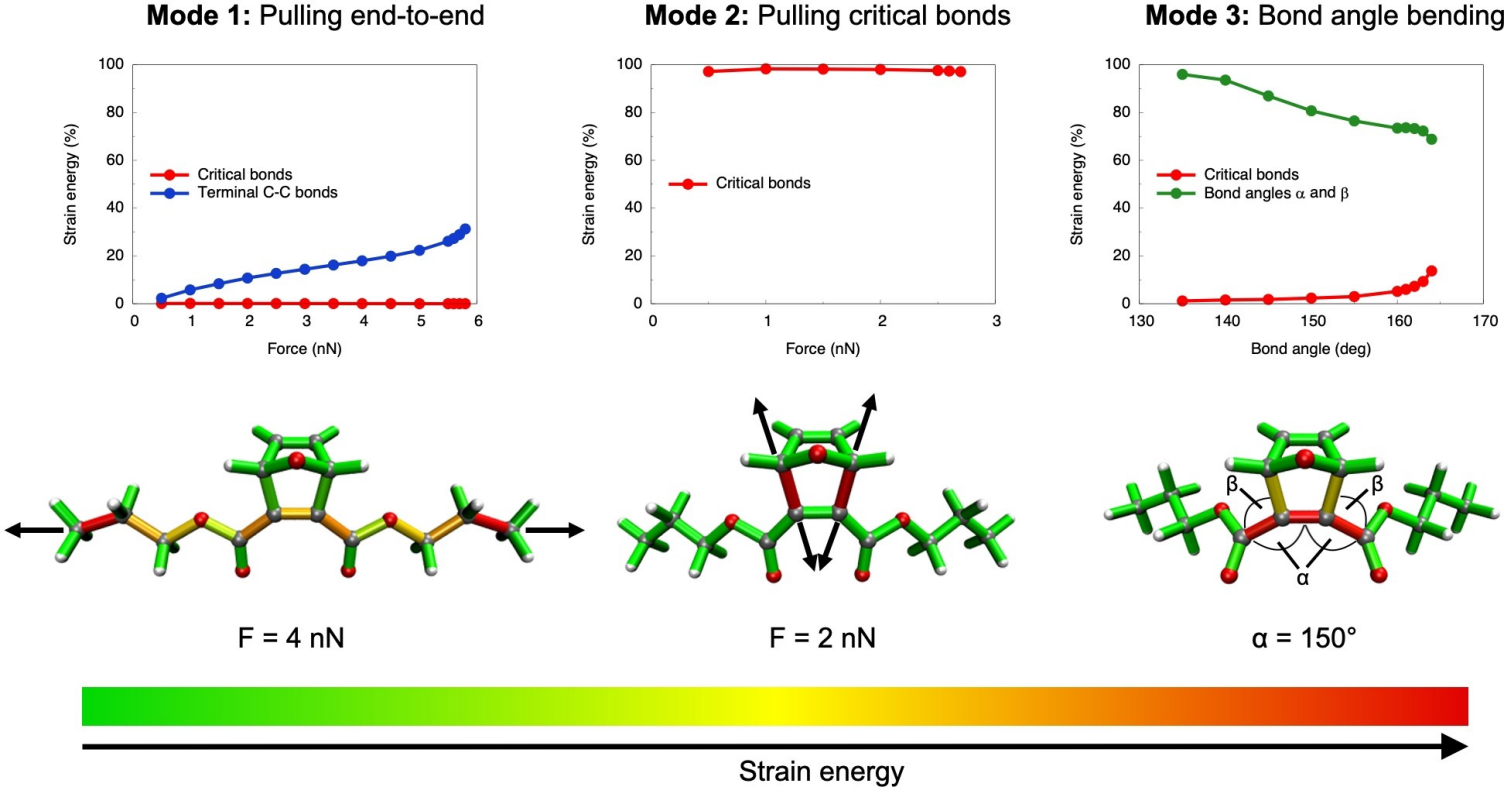
Distribution of strain energy among selected internal coordinates of a flex‐activated mechanophore that is mechanically deformed *via* three different modes, as calculated with the JEDI analysis.^[50–52]^ “Critical bonds” denote the carbon‐carbon bonds that need to be ruptured for a successful retro‐[4+2] cycloaddition. Color‐coded representations of strain energy at selected forces (modes 1 and 2) or bond angles *α* (mode 3) were derived by mapping strain values in bonds, bendings and torsions onto the involved covalent bonds.

Mode 2, on the other hand, constitutes a very efficient way of achieving flex‐activation, which agrees with chemical intuition. Throughout the entire stretching coordinate, the critical bonds store almost 100 % of the strain energy in the system and the retro‐[4+2] cycloaddition occurs efficiently if both bonds are being pulled with a force of 2.8 nN, since no strain is wasted on other parts of the molecule.

In a mechanical pulling experiment, however, it is far from straightforward to trigger flex‐activation *via* mode 2, since the critical bonds need to be stretched in an isolated manner. A more realistic scenario is the bending of the bond angles in the main polymer chain that are adjacent to the critical bonds (mode 3). If the bond angles *α* (cf. Figure 1) are displaced from their initial values of 131.7° to 164.1°, the critical bonds rupture and the retro‐[4+2] cycloaddition occurs. The bond angle bendings store most strain energy throughout the entire deformation coordinate and pass on some of it to the critical bonds, leading to efficient rupture of the latter at 164.1°. Hence, the bond angles in the polymer main chain that are adjacent to the critical bonds act as work funnels that localize the strain energy in the region of the scissile bonds. An analogous effect was found for torsions, which facilitate the rupture of knotted polymer chains.^[40]^

Interestingly, immediately before bond rupture the strain energies stored by the critical bonds are very similar in mode 2 (15.2 kcal/mol) and mode 3 (17.9 kcal/mol), signifying the same degree of weakening of these bonds in both modes.

The unsuitability of mode 1 for flex‐activation is emphasized when combining it with mode 2 (Figure 2, top panel). The rupture force of the critical bonds when pulling them directly decreases only slightly when simultaneously stretching the molecule end‐to‐end: It decreases from 2.8 nN in the absence of end‐to‐end stretching to 2.3 nN shortly before the terminal carbon‐carbon bonds are ruptured. Since a significant amount of force is still required to achieve the retro‐[4+2] cycloaddition, end‐to‐end stretching barely diminishes the strength of the critical bonds. When combining mode 2 and mode 3, on the contrary, the rupture forces of the critical bonds decrease monotonically (Figure 2, bottom panel). This again demonstrates the weakening of the critical bonds by adjacent bond angle bendings. Decreasing the bond angles *α* form their optimal value (131.7°) leads to an increase in the rupture force of the critical bonds.

**Figure 2:**
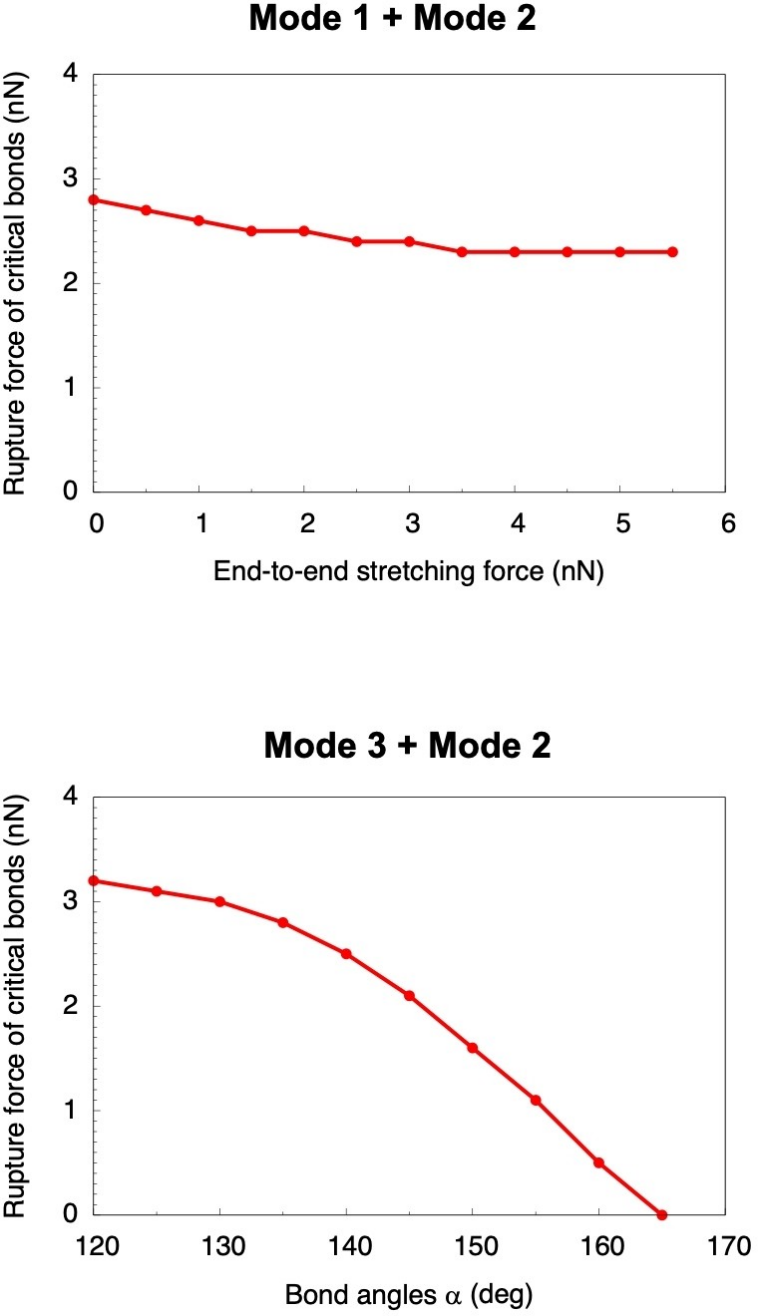
Rupture forces of the critical bonds *via* mode 2 when simultaneously stretching the molecule end‐to‐end (mode 1, top panel) or constraining the bond angles *α* (mode 3, bottom panel).

In an effort to identify the reason why adjacent bond angle bendings weaken the critical bonds, the Quantum Theory of Atoms in Molecules (QTAIM) was applied.^[53]^ Increasing the adjacent bond angles leads to a decrease of electron density in the critical bonds (Figure 3). Conversely, the electron density in other covalent bonds in the molecule increases, two of which are singled out in Figure 3 as well. The mechanism by which adjacent bond angle displacements weaken the critical bonds and thus facilitate flex‐activation is therefore a complex phenomenon that involves the rearrangement of electron density within the furan ring that is being created by the retro‐[4+2] cycloaddition as well as in certain parts of the polymer chain. Interestingly, the electron density in the critical bonds hardly changes during deformation *via* modes 1 and 2 (cf. Supporting Information, Figure S1), showing that the rearrangement of electron density is a unique feature of deformation mode 3.

**Figure 3:**
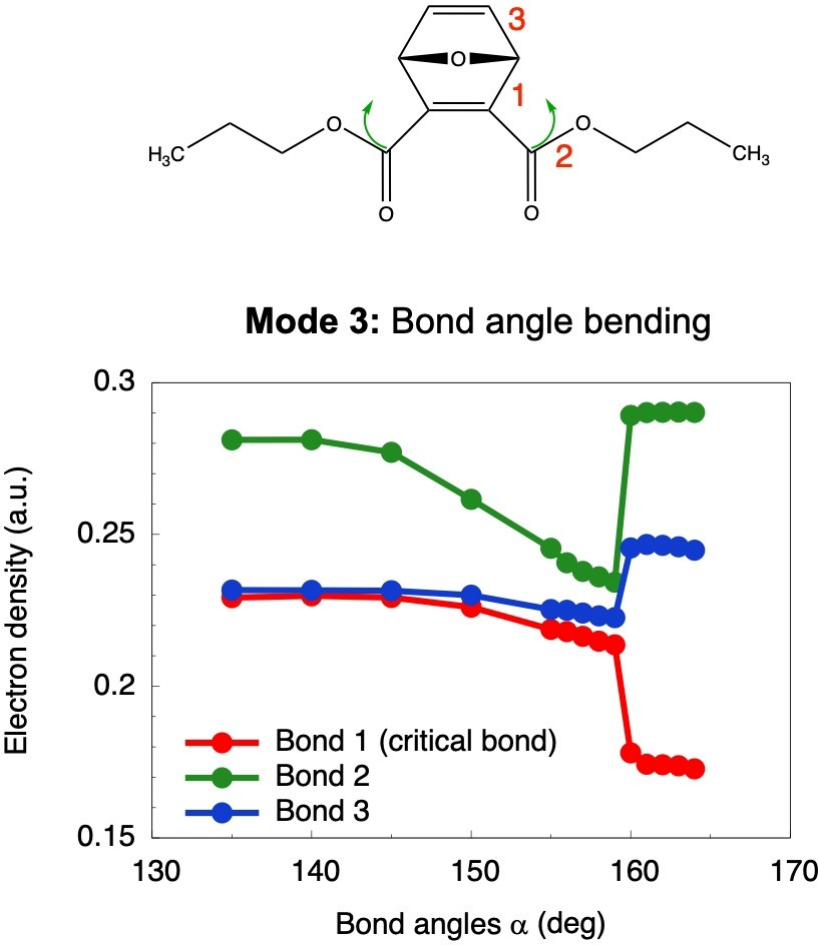
Progression of electron density in selected bonds (1*−*3) when constraining the bond angles *α* to different values.

Nevertheless, when stretching a polymer experimentally with the aim of activating mechanophores, an intuitive approach includes stretching the polymer end‐to‐end according to deformation mode 1. One could hypothesize that random thermal oscillations during the experiment lead to a weakening of the critical bonds in a way similar to deformation modes 2 and 3, thus still achieving flex‐activation. To elucidate whether random thermal oscillations in a mechanochemical pulling setup lead to flex‐activation, steered Born‐Oppenheimer Molecular Dynamics (BOMD) simulations with a force‐loading rate of 2.5 *⋅* 10^12^ nN at four different temperatures (300 K, 600 K, 900 K, 1200 K) were carried out (Figure 4). To account for sufficient sampling of random initial velocities, ten trajectories for each temperature were calculated. Flex‐activation was, however, never observed in any simulation. Instead, each trajectory led to rupture of the terminal methyl groups, which, on average, occurred earlier in the simulations if the temperature was higher. While a higher temperature led to increased oscillations of the critical bonds, this weakening influence was insufficient to initiate the retro‐[4+2] cycloaddition.

**Figure 4:**
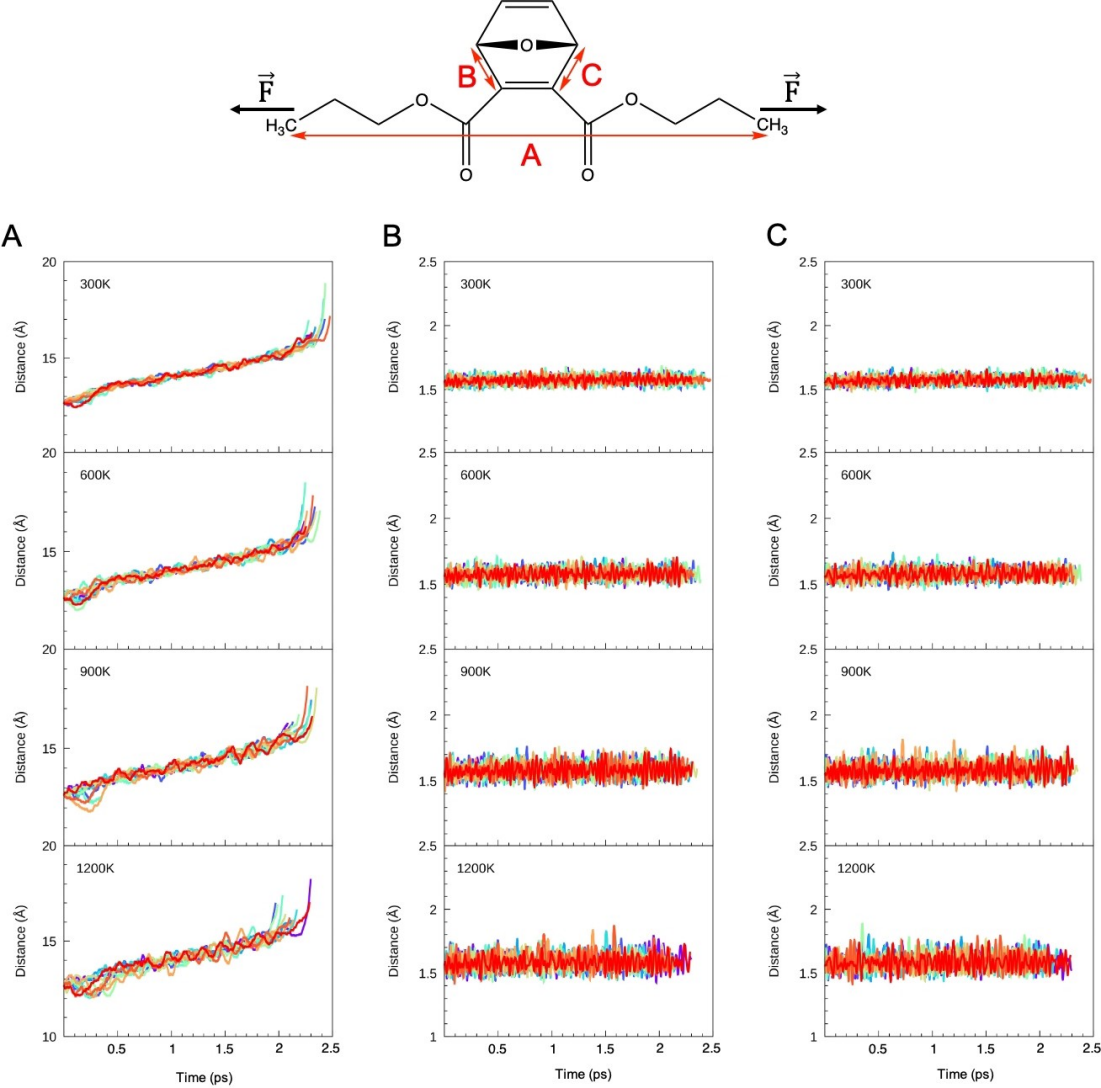
Progression of the end‐to‐end‐distance (A) and the lengths of the critical bonds (B and C) when pulling the molecule apart at the methyl groups during steered Born‐Oppenheimer Molecular Dynamics simulations at different temperatures. Each colored line corresponds to one simulation.

The results presented here demonstrate that the retro‐[4+2] cycloaddition in the investigated flex‐activated mechanophore cannot be triggered by mere end‐to‐end pulling of the main polymer chain. This limits the applicability of flex‐activated mechanophores in mechanochemical pulling experiments, especially in ultrasound baths. The question remains how the activation efficiency in such experiments can be increased. Since deformation modes 2 and 3 have been identified as efficient ways of triggering flex‐activation, we propose using an infrared laser to excite certain normal modes that either lead to a stretching of the critical bonds or to a bending of the bond angles adjacent to them in the polymer chain. Although normal modes are typically delocalized, using the JEDI analysis examples of promising normal modes have been identified (cf. Supporting Information, Figure S2). We hypothesize that tuning an infrared laser to the wavelengths that are necessary to excite these modes during mechanical deformation of polymers including flex‐activated mechanophores will lead to an increase in the rate of flex‐activation and the more efficient release of small molecules. However, it must be noted that the bond angles *α* and *β* need to be displaced significantly to achieve flex‐activation and that it is unknown whether the vibration of a single normal mode is enough to achieve this deformation. Rather, the combination of several normal modes that involve bond angle bendings as well as the stretching of the critical bonds is the most promising approach.

In the future we plan to apply our methodology to a recently proposed mechanophore based on anthracene,^[54]^ for which flex‐activation was tested but remained unsuccessful, with the aim of determining the optimal conditions (e. g. the pulling/deformation setup) for successful flex‐activation. Moreover, we plan to apply quantum mechanochemical models of pressure[^55^, ^56^, ^57^, ^58^] to test the efficiency of flex‐activation in polymers, since experiments on flex‐activated mechanophores were performed under compression.[^24^, ^25^, ^26^] It will be insightful to juxtapose the influences of hydrostatic pressure and mechanical forces deforming the systems, which can be assumed to play a role in the process of flex‐activation.

## Conflict of interest

The authors declare no conflict of interest.

## Supporting information

As a service to our authors and readers, this journal provides supporting information supplied by the authors. Such materials are peer reviewed and may be re‐organized for online delivery, but are not copy‐edited or typeset. Technical support issues arising from supporting information (other than missing files) should be addressed to the authors.

Supplementary
