# Supplementary material for: The Mechanism of Flex‐Activation in Mechanophores Revealed By Quantum Chemistry
Source: Chemphyschem. 2020 Oct 7;21(21):2402–6. doi: 10.1002/cphc.202000739 (PMC7702058; doi:10.1002/cphc.202000739)
Supplement: Supplementary file 1 — Supplementary [file CPHC-21-2402-s001.pdf]

ChemPhysChem

Supporting Information

The Mechanism of Flex-Activation in Mechanophores Revealed By Quantum Chemistry

Lennart J. Mier, Gheorghe Adam, Sourabh Kumar, and Tim Stauch*

Contents

1 Computational Details **2**

2 Electron Density for Deformation Modes 1 and 2 **3**

3 Normal Mode Analysis **4**

4 References **5**

1 Computational Details

All calculations using the External Force is Explicitly Included (EFEI)[1–3] and the COnstrained Geometries simulate External Forces (COGEF)[4,5] approaches were carried out with the Q-Chem 5.2.1 program package.[6] Since mechanical forces can be applied to a molecule directly in the EFEI method, this approach has some clear technical advantages over COGEF for deformation modes 1 and 2.[7,8] However, the use of COGEF is necessary for mode 3, since isolated bond angle bending can only be achieved with COGEF. Rupture forces of the C_s-symmetric model system in deformation modes 1 and 2 were determined iteratively with a resolution of 100 pN. Similarly, the bond angles α that are displaced for flex-activation (deformation mode 3) were determined with a resolution of 0.1°. Density Functional Theory (DFT)[9,10] at the PBE[11]/cc-pVDZ[12] level of theory was used in all calculations. This choice was made due to the lack of experimental data against which to benchmark and because the results calculated with PBE/cc-pVDZ fall within the range of other established computational methods (cf. Table S1).

The Judgement of Energy DIstribution (JEDI) analysis[13–15] was applied to determine the distribution of strain energy among the internal coordinates of the molecule, based on the deformed geometries generated *via* EFEI and COGEF. Color-coded representations were created with VMD 1.9.3[16] by mapping the strain energies in the bond lengths, bond angles and dihedral angles onto the covalent bonds involved in the particular internal coordinate.

The electron densities in the bond critical points were calculated based on the Quantum Theory of Atoms In Molecules (QTAIM)[17] using the Multiwfn 3.7 program package.[18] The wavefunctions required for these calculations were generated with Q-Chem.

Steered Born-Oppenheimer Molecular Dynamics (BOMD) simulations were carried out with Q-Chem 5.2. A time step of 10 a.u. (0.242 fs) was adjusted. The end-to-end stretching force was increased by 250 pN each 413 time steps, i.e. roughly each 0.1 ps. Temperatures of 300K, 600K, 900K and 1200K

were adjusted and ten simulations were carried out for each temperature. Initial velocities were sampled randomly from a Maxwell-Boltzmann distribution. The trajectories were run until bond rupture occurred, which was accompanied by SCF convergence failure. PBE/cc-pVDZ was used as the electronic structure method.

Functional	cc-pVDZ	cc-pVTZ
BP86[19,20]	163.4	161.2
B97[21]	165.5	164.2
PBE[22]	164.1	162.7
BLYP[23,24]	161.0	160.0
B3LYP[23–25]	164.3	162.7
BHHLYP[23–25]	167.5	166.5
PBE0[22,26]	167.2	166.1

Table S1: Bond angles α that lead to flex-activation in the model system, calculated with the COGEF approach at different levels of theory.

2 Electron Density for Deformation Modes 1 and 2

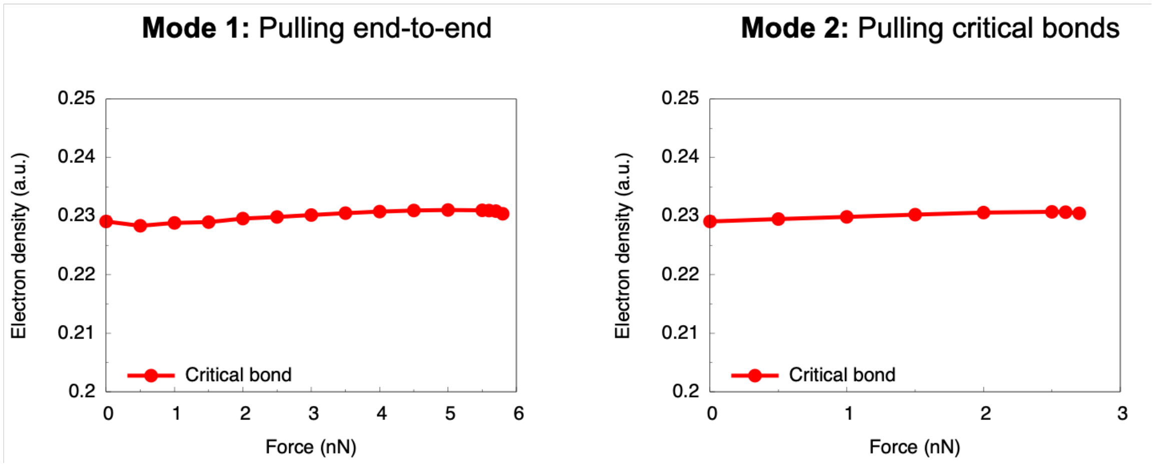

Figure S1: Progression of electron density in each critical bond when applying pulling forces to the flex-activated mechanophore *via* deformation modes 1 and 2.

3 Normal Mode Analysis

For deformation modes 2 and 3, which lead to a successful retro-[4+2] cycloaddition reaction of the flex-activated mechanophore considered in this work, the JEDI analysis in normal modes[13,14] was carried out to determine normal modes that precondition the critical bonds for rupture. Pulling the critical bonds apart directly (mode 2) of course leads to an elongation of these bonds. Using the JEDI analysis it was found that a plethora of normal modes involve an elongation of the critical bonds and therefore store strain energy, two of which are shown in Figure S2. At a stretching force of 2 nN, e.g., normal modes A and B store 11% and 10% of the strain energy, respectively. Applying infrared radiation with the energies appropriate to excite these (and other) normal modes might weaken the critical bonds and thus lead to an increase in the rate of the retro-[4+2] cycloaddition reaction.

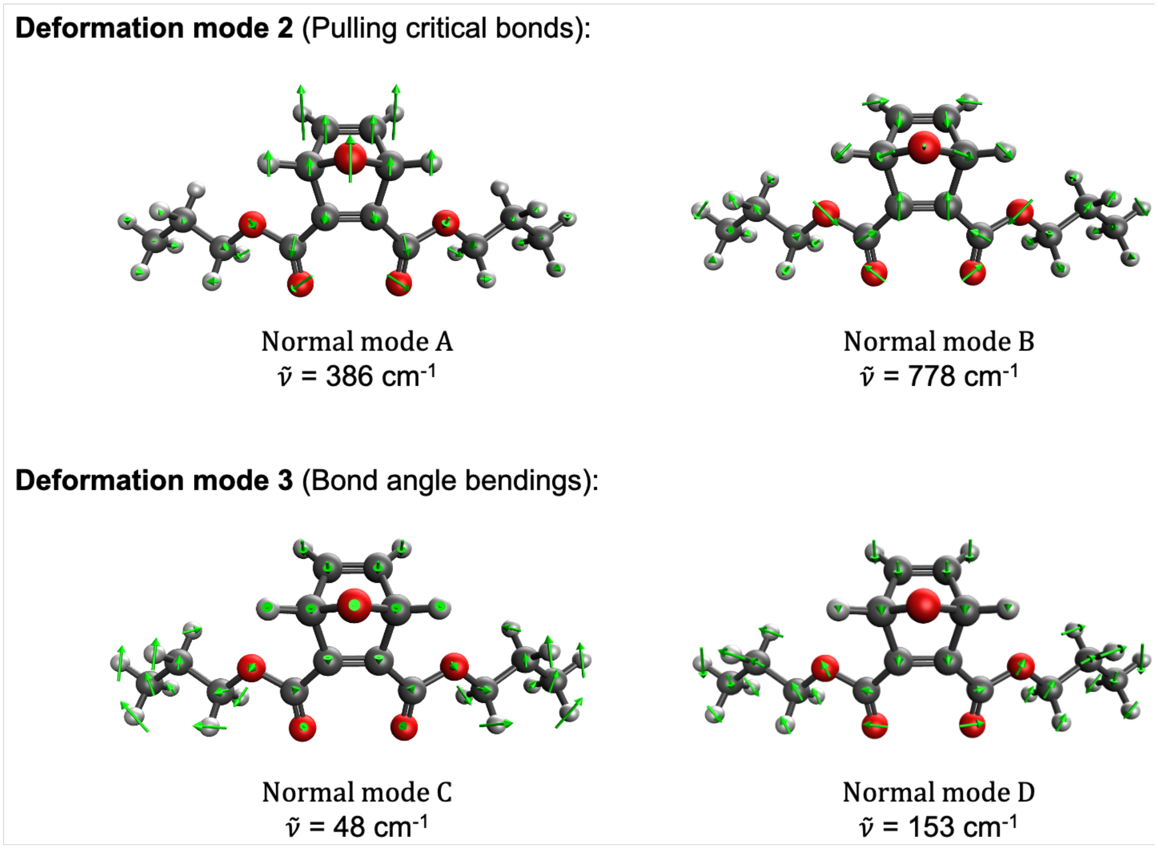

Figure S2: Two selected normal modes for deformation modes 2 and 3, respectively, that either lead to an elongation of the critical bonds (mode 2) or a displacement of the bond angles α (mode 3).

Since normal modes are rectilinear, they describe inherently curvilinear motions like bond angle bendings only poorly.[14] As a result, the error of the harmonic approximation in the JEDI analysis becomes quite large, so that, in the case of deformation mode 3, only minor displacements of the bond angle α were considered. If α is constrained to 135°, the error of the harmonic approximation is only 12%. Normal modes C and D store 21% and 28% of the harmonic strain energy in this case, signifying that a vibration of these modes is beneficial for the retro-[4+2] cycloaddition reaction.

4 References

[1] M. T. Ong, J. Leiding, H. Tao, A. M. Virshup and T. J. Martínez, *J. Am. Chem. Soc.*, 2009, **131**, 6377–6379.

[2] J. Ribas-Arino, M. Shiga and D. Marx, *Angew. Chem. Int. Ed.*, 2009, **48**, 4190–4193.

[3] K. Wolinski and J. Baker, *Mol. Phys.*, 2009, **107**, 2403–2417.

[4] M. K. Beyer, *J. Chem. Phys.*, 2000, **112**, 7307–7312.

[5] E. A. Nikitina, V. D. Khavryutchenko, E. F. Sheka, H. Barthel and J. Weis, *J. Phys. Chem. A*, 1999, **103**, 11355–11365.

[6] Y. Shao, Z. Gan, E. Epifanovsky, A. T. B. Gilbert, M. Wormit, J. Kussmann, A. W. Lange, A. Behn, J. Deng, X. Feng, D. Ghosh, M. Goldey, P. R. Horn, L. D. Jacobson, I. Kaliman, R. Z. Khalliulin, T. Kuś, A. Landau, J. Liu, E. I. Proynov, Y. M. Rhee, R. M. Richard, M. A. Rohrdanz, R. P. Steele, E. J. Sundstrom, H. L. Woodcock, P. M. Zimmerman, D. Zuev, B. Albrecht, E. Alguire, B. Austin, G. J. O. Beran, Y. A. Bernard, E. Berquist, K. Brandhorst, K. B. Bravaya, S. T. Brown, D. Casanova, C.-M. Chang, Y. Chen, S. H. Chien, K. D. Closser, D. L. Crittenden, M. Diedenhofen, R. A. DiStasio, H. Do, A. D. Dutoi, R. G. Edgar, S. Fatehi, L. Fusti-Molnar, A. Ghysels, A. Golubeva-Zadorozhnaya, J. Gomes, M. W. D. Hanson-Heine, P. H. P. Harbach, A. W. Hauser, E. G. Hohenstein, Z. C. Holden, T.-C. Jagau, H. Ji, B. Kaduk, K. Khistyaev, J. Kim, J. Kim, R. A. King, P. Klunzinger, D. Kosenkov, T. Kowalczyk, C. M. Krauter, K. U. Lao, A. D. Laurent, K. V. Lawler, S. V. Levchenko, C. Y. Lin, F. Liu, E. Livshits, R. C. Lochan, A. Luenser, P. Manohar, S. F. Manzer, S.-P. Mao, N. Mardirossian, A. V. Marenich, S. A. Maurer, N. J. Mayhall, E. Neuscamman, C. M. Oana, R. Olivares-Amaya, D. P. O'Neill, J. A. Parkhill, T. M. Perrine, R. Peverati, A. Prociuk, D. R. Rehn, E. Rosta, N. J. Russ, S. M. Sharada, S. Sharma, D. W. Small, A. Sodt, T. Stein, D. Stück, Y.-C. Su, A. J. W. Thom, T. Tsuchimochi, V. Vanovschi, L. Vogt, O. Vydrov, T. Wang, M. A. Watson, J. Wenzel, A. White, C. F. Williams, J. Yang, S. Yeganeh, S. R. Yost, Z.-Q. You, I. Y. Zhang, X. Zhang, Y. Zhao, B. R. Brooks, G. K. L. Chan, D. M. Chipman, C. J. Cramer, W. A. Goddard III, M. S. Gordon, W. J. Hehre, A. Klamt, H. F. Schaefer, M. W. Schmidt, C. D. Sherrill, D. G. Truhlar, A. Warshel, X. Xu, A. Aspuru-Guzik, R. Baer, A. T. Bell, N. A. Besley, J.-D. Chai, A. Dreuw, B. D. Dunietz, T. R. Furlani, S. R. Gwaltney, C.-P. Hsu, Y. Jung, J. Kong, D. S. Lambrecht, W. Liang, C. Ochsenfeld, V. A. Rassolov, L. V. Slipchenko, J. E. Subotnik, T. Van Voorhis, J. M. Herbert, A. I. Krylov, P. M. W. Gill and M. Head-Gordon, *Mol. Phys.*, 2014, **113**, 184–215.

[7] T. Stauch and A. Dreuw, *Chem. Rev.*, 2016, **116**, 14137–14180.

[8] G. S. Kochhar, G. S. Heverly-Coulson and N. J. Mosey, *Top. Curr. Chem.*, 2015, **369**, 37–96.

[9] P. Hohenberg and W. Kohn, *Phys. Rev.*, 1964, **136**, 864–871.

[10] W. Kohn and L. J. Sham, *Phys. Rev.*, 1965, **140**, 1133–1138.

[11] J. P. Perdew, K. Burke and M. Ernzerhof, *Phys. Rev. Lett.*, 1996, **77**, 3865–3868.

[12] T. H. Dunning, *J. Chem. Phys.*, 1989, **90**, 1007–1023.

[13] T. Stauch and A. Dreuw, *J. Chem. Phys.*, 2014, **140**, 134107.

[14] T. Stauch and A. Dreuw, *J. Chem. Phys.*, 2015, **143**, 074118.

[15] T. Stauch and A. Dreuw, *Acc. Chem. Res.*, 2017, **50**, 1041–1048.

[16] W. Humphrey, A. Dalke and K. Schulten, *J. Mol. Graphics*, 1996, **14**, 33–38.

[17] R. F. Bader, *Acc. Chem. Res.*, 1985, **18**, 9–15.

[18] T. Lu and F. Chen, *J. Comput. Chem.*, 2012, **33**, 580–592.

[19] J. P. Perdew, *Phys. Rev. B*, 1986, **33**, 8822–8824.

[20] A. D. Becke, *J. Chem. Phys.*, 1988, **88**, 1053–1062.

[21] A. D. Becke, *J. Chem. Phys.*, 1997, **107**, 8554–8560.

[22] J. P. Perdew, K. Burke and M. Ernzerhof, *Phys. Rev. Lett.*, 1997, **78**, 1396.

[23] A. D. Becke, *Phys. Rev. A*, 1988, **38**, 3098–3100.

[24] C. Lee, W. Yang and R. G. Parr, *Phys. Rev. B*, 1988, **37**, 785–789.

[25] A. D. Becke, *J. Chem. Phys.*, 1993, **98**, 1372–1377.

[26] C. Adamo and V. Barone, *J. Chem. Phys.*, 1999, **110**, 6158–6170.
